# Adrenergic Mechanism in the Control of Endothelial Function

**Published:** 2011-10-17

**Authors:** Daniela Sorriento, Bruno Trimarco, Guido Iaccarino

**Affiliations:** 1 Department of Internal Medicine of Federico II University of Naples, Napoli, Italy; 2 School of Medicine of University of Salerno, Baronissi (SA), Italy

## Abstract

There is considerable evidence that many disease are associated with endothelial dysfunction and reduced nitric oxide production such as hypertension, obesity, dyslipidemias, diabetes, heart failure, atherosclerosis. Notably these conditions are also characterized by alteration in the adrenergic tone. Whether these two mechanisms are just epiphenomenal each other or there is a functional link, it is still to be established. A starting ground to establish this issue is that vascular endothelium plays an important role in the function of cardiovascular system and that adrenergic receptors on endothelial cells contribute to the regulation of vasomotor tone. The aim of this excerpt is to review current knowledge on the physiology of endothelial adrenergic receptors to contribute to the basis for newer and better approaches to endothelial dysfunction in the setup of cardiovascular conditions.

## Introduction

The endothelium controls several vascular functions, including vasculature tone and permeability, thrombosis, hemostasis and angiogenesis^1^–^4^. It is noteworthy that all these functions can be regulated by the activation of receptors and often the same receptor can activate multiple endothelial functions. The adrenergic system is the major regulator of cardiac and vascular function and of endothelial vasorelaxation by means of α and β adrenergic receptors activation. The adrenergic receptors (ARs) are part of a large family of G protein coupled receptors (GPCR) which mediate the functional effects of catecholamines like epinephrine and norepinephrine. The ARs family includes three β (β_1_, β_2_, β_3_), three α_1_ (α_1A_, α_1B_, α_1D_) and three α_2_ (α_2A_, α_2B_, α_2C_) receptor subtypes. These receptors actively participate to the release of nitric oxide (NO) in order to regulate endothelial function^5^. NO plays a crucial role in endothelium homeostasis, with important vasodilatory, anti-thrombotic and anti-atherogenic properties. NO mediates most of the endothelial functions: it has been invoked as a mechanism in vasorelaxation, endothelium permeability and neoangiogenesis^3^. NO in the endothelium is constitutively produced by the endothelial NO synthase, eNOS^6^. This latter is then further activated through calcium levels 7 and phosphorylation of various serine residues by a number of protein kinases 8, 9. Indeed, it has been demonstrated that NO is activated by means of the PI3K pathway in response to the stimulation of tyrosine kinase 10,11.

The impaired ability of vascular endothelium to stimulate vasodilation is referred to as “Endothelial Dysfunction” and the major cause is the decreased bioavailability of NO in different conditions which can be due to various mechanisms: reduced eNOS expression, altered NO production and increased NO catabolism. Endothelial dysfunction plays a key role in the development of cardiovascular disease such as hypertension, type 2 diabetes and heart failure. The identification of the underlying pathogenic mechanisms will lead to the discovery of newer and more potent tools to treat such diseases. On this issue, endothelial dysfunction has been associated to signal transduction abnormalities observed in hypertension. In particular, adrenergic vasorelaxation has been demonstrated to be impaired in hypertensive patients, probably due to the presence of increased desensitization and impaired signalling of βAR. Adrenergic receptors on endothelium have been longely not considered functional to the regulation of the vascular tone. On the contrary, it is possible to identify very specific roles for such receptors in several endothelial function. This review will summarize the effects of adrenergic receptors on endothelial functions, focusing on modulation of NO synthesis and angiogenesis.

## α adrenergic receptors

αAR are GPCRs that couple to Gαq protein. The Gαq subunit is a primary activator of phospholipase C (PLC). Activation of PLC promotes the cleavage of the inositol substrate phosphatidyl-inositol 4,5 bisphosphate (PIP2) to yield diacylglycerol (DAG) and inositol 1,4,5-trisphosphate (IP3). DAG and IP3 promote the activation of a protein kinase C (PKC). α_1_AR can also activate specific adenylate (adenylyl) cyclases (AC) leading to an increase in cAMP levels. The activation of specific PLCs and ACs requires a complex balance of signals from G-proteins, especially the Gα subunits, within specific cell contexts. DAG and cAMP are second messengers that affect a wide array of cell signaling pathways and responses.

### 1. α_1_AR and Nitric oxide

Several reports 12, 13 have produced evidence for the functional presence of vasorelaxant α_1_AR in the brachial and pulmonary arteries isolated from the rabbit and rat, respectively. According to these reports, the pharmacological stimulation of α_1_AR located on endothelial cells, is able to generate NO, whereas the stimulation of α_2_AR releases a relaxing prostanoid^12^, ^13^. Filippi demonstrated that nanomolar concentrations of phenylephrine, which are devoid of any contractile effect, induced a slight endothelium-dependent vasorelaxation in the rat mesenteric vascular bed through the stimulation of α_1D_AR, located on endothelial cells, which act through phospholipase C stimulation, followed by IP1 generation, and nitric-oxide synthase activation. Conversely, the increase in perfusion pressure induced by micromolar concentrations of phenylephrine is attributable to the stimulation of α_1A_AR^14^.

### 2. α_1_AR and angiogenesis

Neo-angiogenesis has long been known to be a highly ordered multistep molecular process under tight regulation by endothelial cells^15^ and closely associated with endothelial cell proliferation and migration and to the capability of these cells to modulate the levels of VEGF, the most important cytokine system involved in the formation of new vessels^16^. A series of biological, chemical, hormonal effectors can interfere with this process. Several data support the notion that α_1_-adrenergic receptor should also be ranked among these agents. Indeed, it has been demonstrated that the α_1A_- and the α_1B_-AR subtypes but not the α_1D_ subtype are expressed in cultured rat aorta endothelial cells. The activation of these α_1_-AR in endothelial cells provide a negative regulation of angiogenesis^17^. Indeed, pharmacological antagonism of α_1_-AR in endothelial cells from WKY rats by doxazosin enhanced, while stimulation of these adrenergic receptors with phenylephrine, inhibited endothelial mechanisms of angiogenesis such as cell proliferation and DNA synthesis, ERK and retinoblastoma protein (Rb) phosphorylation, cell migration and tubule formation^17^. A similar phenotype can be observed *in vivo*, since an increased α_1_-adrenergic receptor density in the ischaemic hindlimb, compared to non-ischaemic hindlimb, suggested an enhanced α_1_-adrenergic receptor tone in the ischaemic tissue. Treatment with doxazosin did not alter systemic blood pressure but enhanced neo-angiogenesis in the ischaemic hindlimb^17^.

### 3. α_2_ARand Nitric oxide

It has been demonstrated that α_2_ adrenergic agonists cause endothelium dependent relaxation, that is reduced or abolished by inhibitors of L-arginine/NO pathway. It depends on the activation of α_2_AR on endothelial cells which stimulates the release of NO, an action that would tend to attenuate vasoconstriction produced by the activation of post-junctional vascular α_1_AR^18^–^20^. The α_2_AR subtype that cause endothelium dependent relaxation belongs to the α_2A/D_ subtype, despite the prominent presence of α_2C_AR (77% of α_2C_ versus 23% of α_2A/D_)^21^. It appears that this ratio may not be constant, since it varies within the vascular bed. Indeed, Bockman demonstrated that in the rat mesenteric artery the α_2_AR is coupled to endothelium dependent NO-mediated relaxations and belongs to the α_2A/D_ subtype appearing in its α_2D_ version 22. It has been demonstrated that endothelium dependent relaxation to α_2_ adrenergic agonists is prevented by pertussis toxin 23–28, suggesting the involvement of G_i_ proteins in the signal transduction from the receptor to the activation of nitric oxide synthase 29, 30. Indeed, α_2_ adrenergic agonists cause activation of G_i_ proteins in endothelial cells and stimulate NO synthase activity 31, 32. Contrary to what expected, cAMP is not involved in the signal transduction pathway for α_2A/D_AR mediated NO formation 22. Indeed, the use of forskolin to oppose α_2_ adrenergic receptor mediated inhibition of cAMP formation in endothelium did not affect the relaxant response to α_2_AR agonists, suggesting that cAMP is not involved in the coupling of α_2_AR to NO. There are physiological modulation of endothelium dependent relaxation to α_2_ adrenergic agonists. Such relaxation is upregulated by chronic increase in blood flow 33 or exercise training 34. Insulin enhances NO mediated vasorelaxation both in animal 25 and human 32 vasculature.

## β-adrenergic receptors

**Figure 1:**
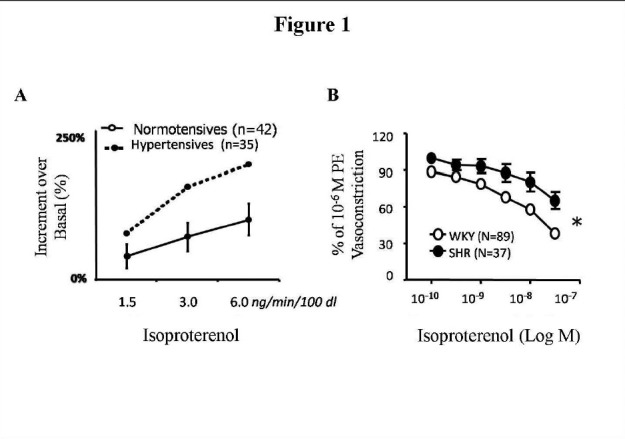
β**AR vasodilation is impaired in hypertension**: A) In hypertensive patients, forearm vasodilation to ISO yielded an increase in forearm blood flow that was significantly lower to that observed in normotensive patients, at each dose of ISO. B) In hypertensive rats SHR, βAR-induced vasorelaxation to ISO in control-treated carotids was significantly impaired compared with that observed in normotensive WKY(* F= 5.756, *p*< 0.01, 2-way ANOVA).

βARs signal by coupling to the stimulatory G protein, Gs, leads to the activation of adenylyl cyclase and accumulation of the second messenger cAMP^35^, ^36^. However, recent studies indicate that under certain conditions βAR, and particularly β_2_AR, can couple to Gi as well as to Gs 37–41. It is now widely accepted that βAR exist on endothelial cells 10, 38, 40, 42 and contribute to the regulation of vasomotor tone. βAR are classically known to be present in the vascular smooth muscle cells (VSMC) where they cause vasodilation. The relative relevance of endothelial VSMC in adrenergic vasodilation is demonstrated by the observation that, in presence of intact endothelium, vasorelaxation to βAR agonist, isoproterenol (ISO), is sensitive to low doses of ISO (10^−10^M-10^−8^M). On the contrary, in absence of endothelium, the vasorelaxation is sensitive to higher doses of ISO (10^−7^M-10^−5^M). This appears to hold true through experimental models (rat or man) and vascular districts (*see* Figure 1).

### 1. β_1_ and β_2_ adrenergic receptors

It is now recognized that βAR located in the endothelium play an important role in the relaxant response to ISO, since the non selective β_1_-and β_2_-adrenergic receptor antagonist propranolol antagonized this relaxant effect^43^, ^44^. However, recent studies carried out in humans, in umbilical veins *in vitro*^10^ or in the forearm *in vivo*^45^, showed that vasorelaxation to ISO is abolished by the selective β_2_AR antagonist ICI-118551 and remains unchanged in the presence of the β_1_AR antagonist CGP-20712, indicating that, as in the vascular smooth muscle cells 46, the endothelial βAR are totally or at least predominantly of the β_2_ subtype 10, 45.

β_2_AR are seven transmembrane receptors coupled through G_s_ proteins to a cAMP dependent intracellular pathway^47^. It has been demonstrated that PKA posphorylation of the third intracellular loop of the β_2_AR increases the affinity of the receptor for G_i_ protein^48^, ^49^. This switch leads to two consequences: first, it decreases the rate of cAMP generation, since G_i_ activation inhibits adenylyl cyclase activity. Second, it increases non cAMP dependent signaling through G_i_, such as activation of the extracellular signal-regulated kinases ERK1/2 and PI_3_K^50^–^54^. Gi coupled receptors have been shown to regulate non-receptor tyrosine kinases, such as SRC, which acts as an intermediate between G_i_ and other molecules like RAS and PI_3_K 53, 55.

### 2. β_2_AR and Nitric oxide

For years it has been given for granted that vascular β_2_AR mediate adrenergic vasorelaxation through direct activation of vascular smooth muscle cells^56^. However, recent data challenge this vision, and show that β_2_AR-dependent vasorelaxation is mediated at least in part, by endothelium through nitric oxide (NO) dependent processes^10^. We have recently demonstrated that the β_2_AR are expressed on endothelial cells (EC) and their stimulation causes endothelial nitric oxide synthase (eNOS) activation^57^. In particular, β_2_AR couple to eNOS and induce NO dependent vasodilation 57. The mechanism of eNOS activation following β_2_AR stimulation is known to be AKT dependent^58^.

Indeed, the activity of eNOS is regulated by both a calcium/calmodulin dependent fashion^59^ and AKT dependent eNOS phosphorylation in Ser 1177 8, 60–63. AKT is primarily activated in response to stimulation of transmembrane receptors with intrinsic tyrosine kinase activity or indirectly coupled to tyrosine kinases or to seven transmembrane G protein-coupled receptor^11^, ^61^, ^64^. Therefore AKT acts as integrator of different signal transduction pathways converging on eNOS, including endothelial β_2_AR receptor^9^, ^58^, ^62^, ^63^, ^65^.

### 3. β_2_AR and angiogenesis

In the endothelium βARs control other important endothelial functions like angiogenesis, that is tightly associated to endothelial cell migration and proliferation 57, 65, 66. We demonstrated that β_2_AR stimulation with ISO and the overexpression of β_2_AR increases endothelial cell proliferation. Moreover, β_2_AR stimulation induces ERK phosphorylation and the MEKK inhibitor, U0126, inhibits β_2_AR induced cell proliferation 66 suggesting that β_2_AR dependent cell proliferation is dependent on ERK activation. We studied post-ischaemic angiogenesis in the hindlimb (HL) of β_2_AR knock-out mice (β_2_AR−/−) in vivo and explored possible molecular mechanisms in vitro. Angiogenesis was severely impaired in β_2_AR−/− mice subjected to femoral artery resection, but was restored by gene therapy with ADβ_2_AR. The proangiogenic responses to a variety of stimuli were impaired in β_2_AR−/− EC *in vitro*^17^. Moreover, removal of β_2_ARs impaired the activation of NFκB, a transcription factor that promotes angiogenesis; ISO did not induce NFκB activation in β_2_AR(−/−) EC^17^. ADβ_p2_AR administration restored β_2_AR membrane density and reinstated the NFκB response to ISO 17. These results suggest that β_2_ARs control angiogenesis through the tight regulation of nuclear transcriptional activity.

### 4.

### 5. α_1_ARand β_2_AR differently regulate neo-angiogenesis

α_1_- and β_2_-adrenergic receptors mediate opposite effects on neo-angiogenesis, comparable to their regulation of the vascular tone. In particular, the α_1_-AR is inhibitory, whereas the β_2_-AR is stimulant to neo-angiogenesis. Interestingly, in ischaemia, the α_1_-AR are upregulated, thus causing a predominance of α_1_-adrenergic receptor signalling over that of β_2_-AR, which is downregulated. Furthermore, in conditions such as hypertension, where the α_1_-AR tone is higher than that of the β_2_-AR, there is also an impairment in neo-angiogenesis 66, 67. It is interesting to note that in the ischaemic hindlimb, α_1_-AR blockade resulted in a normalization of β_2_-AR density together with improved neo-angiogenesis. α_1_-AR upregulation, in particular, might be a regulatory mechanism aimed at preventing excessive angiogenesis. This upregulation might be triggered by ischaemia, through regulatory sequences within the gene promoter, which have been demonstrated for both the α_1A_- and α_1B_-adrenergic receptor^68^, ^69^.

### 6. β_3_ adrenergic receptors

In rat thoracic aorta, Trochu showed that β_3_AR are mainly located on endothelial cells and act in conjunction with β_1_AR and β_2_AR to mediate relaxation through activation of NO synthase pathway and subsequent increase in tissue cyclic GMP content and is reduced by endothelium removal or in presence of L-NMMA 70. This β_3_AR mediated aorta relaxation seems to be independent of G_i_ proteins stimulation, since the blockage of G_i_ protein by PTX does not modify β_3_AR agonists induced relaxation. On the contrary, selective potassium channels blockers of K (Ca), K (ATP) and K (v) decreased β_3_AR agonists induced relaxation. So it appears that this effect results from the activation of several potassium channels, K (Ca), K (ATP) and K (v) 71.

## Pathological implications

It was reported that noradrenaline-induced release of nitric oxide is enhanced in mineralcorticoid hypertension 72 indicating that α_2_AR may play an important role in the regulation of vascular tone not only in physiological but also in pathological conditions. The implications of impaired βAR signalling in the pathophysiology of several cardiovascular disorders has been studied in animals and humans. Data from these studies indicate that changes in βAR function are induced by heart failure 73, 74 and hypertension 75, 76. Moreover, alteration in βAR function were found also with physiological aging 77, 78, due to receptor downregulation and desensitization. Exercise restored the impaired signalling and βAR dependent vasorelaxation^79^. We and others have observed that impaired βAR signalling may account for dysfunctional βAR vasorelaxation in hypertension. In this condition, β_2_AR overexpression in hypertensive rat carotids corrects impaired vasorelaxation to βAR stimulation to levels similar to those seen in normotensive rats^57^. We proved that impaired endothelium dependent vasorelaxation in spontaneously hypertensive rats (SHR) can be corrected by increasing the signal transduction pathways leading to nitric oxide synthase activation 80. In particular, since eNOS is activated in response to phosphorylation by AKT and impaired AKT activity is involved in endothelial dysfunction, AKT overexpression should result in the correction of impaired phenotype. Indeed, insulin and ISO cause AKT membrane localization and this subcellular localization is impaired in SHR. AKT overexpression, through means of adenovirus mediated AKT gene transfer to the endothelium, increases the amount of AKT localized to the membrane and corrects impaired NO release and endothelium dependent vasodilation to agonists of both the GPCR and tyrosine kinase (TK) dependent pathways.

## Conclusions

In the last years great advances have been made in the study of adrenergic receptors signaling and function in the endothelium also thanks to the development of new technologies. Indeed, genetic mouse models have significantly improved our understanding of the mechanisms of action of specific drugs *in vivo*. The ability to induce transgene expression at defined times or in defined tissues is an important goal as well as the ability to induce or repress the expression of endogenous genes in a developmental or tissue specific fashion. Indeed, deletion of the genes encoding for adrenergic receptor subtypeshas helped to identify the specific subtypes whichmediate *in vivo* effects of specific drugs. Thus, the combination of molecular biological, genetic, and pharmacological techniques greatly facilitates our understanding of adrenergic receptor function *in vivo*, and in turn leads to more effective and specific therapeutic treatment in humans. βARs, for instance, are already target of therapeutic intervention in many diseases: βAR stimulation in asthma and obesity or βAR blocking in hypertension and coronary insufficiency. In conclusion, giving the importance of endothelial function in most physiological and pathological conditions, it is clear that the increasing knowledge of adrenergic receptors function in the endothelium is helpful for future progresses in clinical application.
